# Oral and anal sex practices among high school youth in Addis Ababa, Ethiopia

**DOI:** 10.1186/1471-2458-12-5

**Published:** 2012-01-04

**Authors:** Amsale Cherie, Yemane Berhane

**Affiliations:** 1 School of Public Health, Addis Ababa University, P.O. Box: 33412, Addis Ababa, Ethiopia; 2 Addis Continental Institute of Public Health, P.O. Box: 26751/1000, Addis Ababa, Ethiopia

**Keywords:** Youth, Sexual and reproductive health, Oral sex, Anal sex, HIV, STIs

## Abstract

**Background:**

Understanding the full range of sexual behaviors of young people is crucial in developing appropriate interventions to prevent and control sexually transmitted infections including HIV. However, such information is meager in developing countries. The objective of this study was to describe oral and anal sex practices and identify associated factors among high school youth.

**Methods:**

A cross-sectional study was conducted among high school youth in Addis Ababa, Ethiopia. A multi-stage sampling procedure was followed to select a representative sample of school youth. The total sample size for this study was 3840. Data were collected using a self-administered questionnaire. Data analysis was guided by the ecological framework.

**Results:**

The overall proportion of people who reported ever having oral sex was 5.4% (190) and that of anal sex was 4.3% (154). Of these 51.6% (98) had oral sex and 57.1% (87) had anal sex in the past 12 months. Multiple partnerships were reported by 61.2% of the respondents who had oral sex and 51.1% of students practicing anal sex. Consistent condom use was reported by 12.2% of those practicing oral sex and 26.1% of anal sex. Reasons for oral and anal sex included prevention of pregnancy, preserving virginity, and reduction of HIV and STIs transmission. Oral sex practice was strongly and significantly associated with perception of best friends engagement in oral sex (AOR = 5.7; 95% CI 3.6-11.2) and having illiterate mothers (AOR = 11.5; 95%CI 6.4-18.5). Similarly, anal sex practice was strongly and significantly associated with favorable attitude towards anal sex (AOR = 6.2; 95%CI 3.8-12.4), and perceived best friends engagement in anal sex (AOR = 9.7; 95%CI 5.4-17.7).

**Conclusion:**

Considerable proportion of adolescents had engaged in oral and anal sex practices. Multiple sexual partnerships were common while consistent condom use was low. Sexual health education and behavior change communication strategies need to cover a full range of sexual practices.

## Background

The sexual behavior of youth is a priority public health concern because of the high prevalence of HIV/AIDS and sexually transmitted infections (STIs) among this age group [1]. It is estimated that nearly 50% of the 35.3 million people who have been infected with the HIV virus acquired the infection before age 25 [2]. A total of 980,000 people were living with HIV/AIDS in Ethiopia in 2007 [3]. Each year an estimated 333 million new cases of curable STIs occurs worldwide [4-6]. Abstinence, being faithful, and consistent condom use (ABC) are the recommended prevention interventions. However, young people are still involved in early sexual practices, have multiple sexual partners and do not use condoms consistently [7-10].

Unprotected vaginal-penile intercourse has been known to be the predominant route for HIV and STIs transmission [2-4,11]. However, it is becoming evident that youth are involved in oral and anal intercourse [12-14]. Although the oral and anal sex behaviors of youth have been researched in the United States (US) for more than two decades [15-18], it was only recently that research evidences in some parts of Africa revealed the practice of oral and anal sex [19].

Studies in the US indicated that between 19.6% and 78% of young people had had oral sex in their life time and, of these only few youth used barrier protection against HIV and STIs [15-17]. In contrast a study conducted in Tanzania identified that 8.1% of young people were involved in oral sex [19]. Although there is a perception among youth that oral sex is risk free, evidence supports that several STIs, including chlamydia, human papillomavirus (HPV), gonorrhea, herpes, hepatitis, and HIV can be transmitted through oral sex [20,21].

Unprotected anal intercourse carries the highest risk of HIV transmission as compared to oral and vaginal sex [22]. It was pointed out that between 3% and 41% of girls and between 7% and 20% of boys reported having engaged in anal sex [23,24]. It was identified that 5.0% of young people in Nigeria and 7.5% of students in Tanzania had anal intercourse [19,25]. The majority of students who reported having had anal sex had multiple sexual partners and most of them have not used condoms during anal intercourse [19,23-25].

Most studies and interventions related to youth sexual behavior focused on vaginal-penile intercourse. Also, sexual activity has been measured on the basis of whether young people have had vaginal intercourse or not [26]. Studies have identified that individual, family, and peer factors predict vaginal-penile intercourse among youth [13,27,28]. However, only few studies have assessed the relationship between these multilevel factors with oral and anal sex.

Although data on the proportion of young people engaged in forced oral and anal sex is lacking, available evidences on vaginal sex suggested that not all sexual experiences of young people are voluntary. Extant evidences indicated that coerced first sex among girls in Sub-Saharan Africa ranged from 32% to 50% [28]. Studies also shown that reported unwanted sex among males ranged from 2.5% to 42% among students in Nigeria [29,30].

Studies on oral and anal sex practices and the associated factors among youth in Ethiopia are scarce. In addition, most studies use individual factors as a predictor to sexual behavior while the sexual behavior of young people is influenced by a multitude of factors. The ecological framework, however, looks into the individual, parental and peer influences on youth sexual behavior. Therefore, understanding the determinants of oral and anal sex using the ecological framework is crucial. Thus, it was the purpose of this study to describe oral and anal sex practices and identify associated factors among high school youth. In effect, this research provides the basis for the designing and implementation of effective preventive interventions that seek to minimize sexual risk behavior and, thereby reducing the incidence of HIV and STIs. This research also seeks to inform clinical practice, education and counseling guidelines.

## Methods

This is a cross sectional study conducted among regularly attending high school youth in Addis Ababa, Ethiopia.

Addis Ababa is the capital city of Ethiopia and has an estimated 3 million population. The youth population (15-24 year) constitutes about 20% of the total city population [31]. There were 70 secondary schools in the city (21 Government, 43 private and 6 foreign community schools) at the time of the study. The total number of high school students in the academic year 2008/2009 was 96571. Of this, 84352 students were attending Government high schools. About 49.0% of the Government and 55.4% of the Non -government school students were females [32].

All high schools primarily established to enroll Ethiopian students in the city of Addis Ababa and students whose age was 15-24 years were included in the study. However, students who were not able to complete the questionnaire without assistance such as the visually impaired were excluded.

The study population was selected from the source population using three-stage sampling. Addis Ababa is administratively divided into 10 Sub Cities. In the first step one high school was selected randomly from each sub-city. The sample size for each selected school was assigned proportionate to the total student population. Then, in the selected schools, from each grade, one section was selected by lottery method. Students from the identified section were selected using a systematic sampling method. The starting number was randomly chosen from the first three in the section roll call. Every third student was then taken until the assigned number was reached.

This study is part of a PhD project. Therefore, the sample size was calculated for the whole dissertation based on level of significance of 95%, power 80%, since the sample selection passed through three stages a design effect of 3, and proportion of condom use among adolescents 50.7%. In addition, 20% allowance was considered for non-response based on the findings from previous school based studies [7]. Based on these the required sample size was found to be 3840 students.

The survey was a paper-and-pencil self-administered questionnaire. The questionnaire was prepared in English and translated into Amharic (the Ethiopian national language). The Amharic questionnaire was pre-tested in schools not selected for the study. Two supervisors with master of public health and ten nurses facilitated the data collection. The research team was trained for 2 days to help them understand the purpose of the study and familiarize themselves with the questions so that they can explain to students, if asked. Data collection in all schools was completed within 1 week to minimize information contamination. The Principal Investigator made both scheduled and unscheduled (surprise) supervisory visits during the data collection. Before commencing the study, official contact with concerned personnel of the City Education Bureau, Zonal Education Bureau, directors of the selected schools and guidance of each school were made by the Principal Investigator. Refreshments were provided for all participants.

The study included items dealing with the dependent variable oral and anal sexual behavior of students and potential independent factors at the individual, family, and peer level factors identified from previous studies [26,27,33,34]. The individual level factors included sex, age, self-esteem, attitude about sex, and educational aspirations. Family factors consisted of parental education level and family structure. Peer level factors comprised of perceived oral and anal sexual experience of the participant's best friends.

To assess the oral and anal sexual activity of students all participants were asked whether they ever had oral or anal sexual intercourse in their lifetime. Yes and no responses were available to be circled by the respondents. Oral sex was defined as "when some-one puts his or her mouth on their partner's penis or vagina or lets their partner put his or her mouth on their penis or vagina". Anal sex means when a man puts his penis in his partner's anus or when one lets their partner insert the penis in their anus".

Self-esteem was measured by Rosenberg's self-esteem scale [35].The scale consisted of ten questions answered on a four point scale-from strongly agree to strongly disagree. Attitude about sex was assessed through two items which asked, how do you feel about teenagers having oral sex and, how do you feel about teenagers having anal sex?" Responses included 1 = Favorable and 2 = Unfavorable.

Data coding, entry, and cleaning was processed using EPI info version 6.4 and the analysis was made using SPSS version 15 statistical package. Analysis was guided by the ecologi cal framework. The coding of open ended questions was made by two persons. Each coder categorized the responses in the same way. Logistic regression models were used to see the association of oral and anal sex and independent variables at the individual, familial, and extra-familial levels. Variables were entered into three blocks. Block 1 contained the individual level variables (sex, age, self-esteem, attitude, and college aspiration). Family structure and parental education were entered next in Block 2. At the extra-familial level (Block 3), peer sexual activity was entered. The three regression models were significant at each of the three levels. The predictors in model 1 accounted for 46% of the variance in sexual activity when variables in model 2 were entered the model's explanatory power slightly increased to 50% of the variance and when model 3 variables were included the explanatory power of the model considerably improved and increased to 56%.

Ethical clearance was secured from the Addis Ababa University, School of Public Health and IRB of the Faculty of Medicine. Persons between 15 and 18 years in Ethiopia are regarded 'Consenting Minors' and can be interviewed without parental consent. Verbal informed consent was obtained from each respondent after explaining the purpose of the study. Participants were assured that participation is voluntary, the information they provide will be kept completely anonymous and confidential. Students were also informed the possibility of opting out at any time if they feel to do so.

## Results

A total of 3840 in school youth aged 15-24 were identified from 10 high schools. Of these 92.5% (3543) of students fully responded to the self administered questionnaire. There was no refusal, but 7.7% (297) of participants responded to less than a quarter of the questions. Thus, their responses were not included in the analysis.

### Socio-demographic characteristics of the study population

The Socio-demographic characteristics of the respondents are depicted in Table 1. From the 3543 respondents, 50.5% (1789) were females and 22.5% (796) were between the age group 15 and 16 years. The mean age of the study population was 17.6 years (SD = 1.5). Over all 55.4% (1964) of the respondents live with both parents. Regarding parental education 17.0% (602) and 26.2% (928) of the respondents' fathers and mothers were without formal education, respectively.

**Table 1:** Socio-demographic Characteristics of 3543 High School Students.

Characteristics	Number (%)
**Sex**	

Female	1789(50.5)

Male	1754(48.5)

**Age group (mean = 17.6**, **SD = 1.5 years)**	

Young adolescents (15-16)	796(22.5)

Older adolescents (17-24)	2747(77.5)

**School Grade**	

Ninth	701(19.8)

Tenth	703(20.3)

Preparatory I	770(21.7)

Prep II	670(18.9)

Vocational	699(19.7)

**Living arrangement**	

Both Parents	1964(55.4)

Other arrangements*	1579(44.6)

**Paternal literacy**	

Illiterate and Non formal	602(17.0)

Formal school	2941(83.0)

**Maternal literacy**	

Illiterate and Non formal	928(26.2)

Formal school	2615(73.8)

Addis Ababa, Ethiopia April, 2009

* Include all not living with both parent

The reported oral sex practices of high school students are shown in Table 2. The overall proportion of those who reported ever having oral sex was 5.3% (190). Of which 13.2% (25) initiated oral sex before the age of 10 and 44.2% (84) had the first oral sex without their consent. The mean age at first oral sex among the study population was 14.6 years (SD = 2.1). Among ever had oral sex; 51.6% (98) of the respondents had reported oral sex in the12 months preceding the survey. Of these 61.2%(60) had more than one oral sex partners, 48.0% (47) received gift for the exchange of oral sex, only 12.2% (12) of them used condom every time they had oral sex, and 80.6%(79) of students had intention to continue having oral sex in the future.

**Table 2:** Oral sexual practices among 3543 high School students in Addis Ababa, Ethiopia

Practices	Number (%)
**Reporting ever having oral sex**	190(5.4)

**Age at first oral sex (mean = **14.6, **SD = 2.1)**	

Below 10 years	25(13.2)

11-15 years	103(54.2)

16-20 years	62(32.6)

**Reporting forced first oral sex**	84(44.2)

**Reporting having oral sex (in the past 12 months)**	98(51.6)

**Number of oral sex partners (in the past 12 months)**	

One	38(38.8)

2-4	35(35.7)

More than four	25(25.5)

**Condom use during oral sex (in the past 12 months)**	

Every time	12(12.2)

Sometime	41(41.8)

Never	45(45.9)

**Received money/gift in exchange for oral sex (past 12 months)**	47(48.0)

**Intend to have oral sex within the next six months**	79(80.6)

Table 3 depicts the anal sex practices of respondents. Overall 4.3% (154) of respondents reported ever having anal sex. Of these 19.5% (30) had their first anal sex before the age of 10 and for the 44.2% (68) the first anal sex was not consensual. The mean age at first anal sex among the study population was 14.8 years (SD = 1.7). About 57.1% (87) of the students reported having anal sex in the last 12 months. Of these only 26.1% (23) used condom consistently during anal sex, and 51.1% (45) of the students reported having more than one anal sex partners, 52.3% (46) had received gift for the exchange of anal sex, and 82.9% (63) of respondents intend to continue having anal intercourse in the future.

**Table 3:** Anal sexual practices among 3543 high School students in Addis Ababa, Ethiopia

Practices	Number (%)
**Reporting ever having anal sex**	154(4.3)

**Age at first anal sex (mean = **14.8, **SD = 1.7)**	

Below 10 years	30(19.5)

11-15 years	65(42.2)

16-20 years	59(38.3)

**Reporting forced first anal sex**	68(44.2)

**Reporting having anal sex (in the past 12 months)**	88(57.1)

**Number of anal sex partners (in the past 12 months)**	

One	43(48.9)

2-4	27(30.7)

More than four	18(20.5)

**Condom use during anal sex (in the past 12 months)**	

Every time	23(26.1)

Sometime	31(35.2)

Never	34(38.6)

**Received money/gift in exchange for anal sex (past 12 months)**	46(52.3)

**Intend to have anal sex within the next six months**	63(82.9)

**Figure 1:**
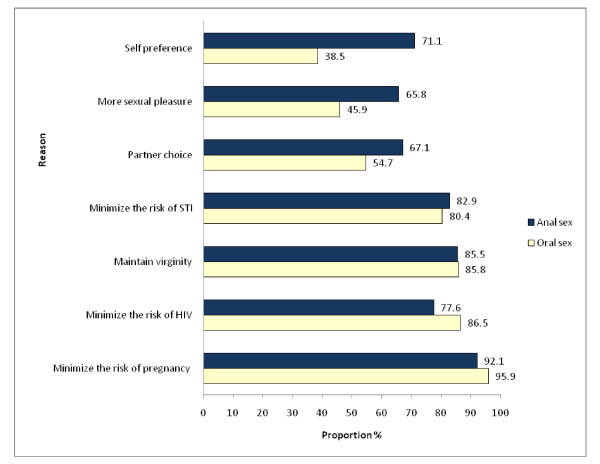
**Reasons for oral and anal sex preference among youth, Addis Ababa, Ethiopia**.

The main reasons given by the respondents for practicing oral sex were preventing pregnancy 95.9% (142), minimizing risk of HIV acquisition 86.5% (128), preserving virginity 85.8% (127), and reducing the risk of STIs transmission 80.4% (119). (See Figure 1).

The predominant reasons reported by the respondents for practicing anal sex were minimizing the risk of pregnancy 92.1% (70), preserving virginity 85.5% (65), minimizing the risk of STIs 82.9% (63), and minimizing the risk of HIV transmission 77.6% (59). (See Figure 1).

Oral sex practice was significantly associated with younger age group (AOR = 3.2; 95%CI: 1.9-5.3), being female (AOR = 1.3; 95%CI 1.1-2.2), having positive attitude about oral sex (AOR = 2.3; 95%CI 1.7-4.5), having low aspirations for college (AOR = 3.1; 95%CI 2.8-5.9), and having low self-esteem (AOR = 2.1; 95%CI 1.7-3.9). (See Table 4).

**Table 4:** Independent correlates of oral sex among 3543 high school students in Addis Ababa, Ethiopia, 2009

Sources of effect	Number (%) Practicing oral sex	Unadjusted OR (95% CI)	Adjusted OR (95% CI)
**Age**			

15-16	79(41.6)	2.6(1.9, 3.6)	3.2(1.9-, 5.3)

17 and above	111(58.4)	Reference	Reference

**Sex**			

Female	101(53.2)	1.1(0.8, 1.5)	1.3(1.1-, 2.2)

Male	89(46.8)	Reference	Reference

**Attitude**			

Positive feeling	52(27.4)	1.3(1.1, 1.7)	2.3(1.7-, 4.5)

Negative feeling	138(72.6)	Reference	Reference

**College Aspiration**			

Low	121(63.7)	3.6(2.1, 4.8)	3.1(2.8-, 5.9)

High	69(36.3)	Reference	Reference

**Self esteem**			

Low	118(62.1)	1.8(1.2, 3.4)	2.1(1.7-, 3.9)

High	72(37.9)	Reference	Reference

**Living arrangement**			

Both parents	78(41.1)	0.5(0.2, 0.7)	0.4(0.2-, 0.9)

Other living arrangements	112(58.9)	Reference	Reference

**Maternal education**			

Illiterate	152(80.0)	13.2(9.4, 17.6)	11.5(6.4-, 18.5)

Literate	38(20.0)	Reference	Reference

**Paternal education**			

Illiterate	47(24.7)	1.6(1.2, 2.9)	1.4(0.9-, 3.2)

Literate	143(75.3)	Reference	Reference

Perception of peer oral sexual activity			

Yes	98(51.6)	4.6(2.8, 7.9)	5.7(3.6-, 11.2)

No	92(48.4)	Reference	Reference

Anal sex was significantly associated with younger age (AOR = 1.7; 95%CI: 1.3-3.1), being male (AOR = 2.9; 95%CI 1.6-4.7), having positive attitude towards anal sex (AOR = 6.2; 95%CI 3.8-12.4), having low college aspirations (AOR = 4.2; 95%CI 2.8-8.1), and having low self-esteem (AOR = 1.6; 95%CI 1.2-3.1). (See Table 5).

**Table 5:** Independent correlates of anal sex among 3543 high school students in Addis Ababa, Ethiopia, 2009

Sources of effect	Number (%) Practicing anal sex	Unadjusted OR (95% CI)	Adjusted OR (95% CI)
**Age**			

15-16	53(34.4)	1.8(1.3, 2.5)	1.7(1.3-, 3.1)

17 and above	101(65.6)	Reference	Reference

**Sex**			

Female	114(74.0)	3.03(1.2, 5.8)	2.9(1.6-, 4.7)

Male	40(26.0)	Reference	Reference

**Attitude**			

Positive feeling	69(44.8)	6.1(3.4, 10.7)	6.2(3.8-, 12. 4)

Negative feeling	85(55.2)	Reference	Reference

**College Aspiration**			

Low	106(68.8)	4.4(2.2, 8.6)	4.2(2.8-, 8.1)

High	48(31.2)	Reference	Reference

**Self esteem**			

Low	87(56.5)	1.5(1.1, 2.9)	1.6(1.2-, 3.1)

High	67(43.5)	Reference	Reference

**Living arrangement**			

Both parents	57(37.0)	0.5(0.1, 0.8)	0.4(0.2-, 0.9)

Other living arrangements	97(63.0)	Reference	Reference

**Maternal education**			

Illiterate	121(78.6)	11.7(5.3, 18.9)	11.6(7.8-, 19.6)

Literate	33(21.4)	Reference	Reference

**Paternal education**			

Illiterate	93(60.4)	8.6(4.8, 12.1)	7.8(5.3-, 14.9)

Literate	61(39.6)	Reference	Reference

**Perception of peer oral sexual activity**			

Yes	105(68.2)	9.6(4.8, 13.2)	9.7(5.4-, 17.7)

No	49(31.8)	Reference	Reference

The study also looked into familial factors that might be associated with anal and oral sex practices. Oral sex practice was less likely among students living with both parents (AOR = 0.4; 95%CI 0.2-0.9). On the other hand students having illiterate mothers were more likely to be involved in oral sex (AOR = 11.5; 95%CI 6.4-18.5). (See Table 4). Likewise, students living with both parents were less likely to have anal sex (AOR = 0.4; 95%CI 0.2-0.9); and students having illiterate mothers were more likely to engage in anal sex (AOR = 11.6; 95%CI 7.8-19.6). Students whose fathers are illiterate were more likely to have anal sex than their counterparts (AOR = 7.8; 95%CI 5.3-14.9) (See Table 5).

Among peer level factors, perception of involvement of best friends in oral sex was significantly associated with oral sex practice (AOR = 5.7; 95%CI 3.6-11.2). (See Table 4). Similarly, students who perceived their best friends are engaged in anal sex were more likely to have anal sexual activity (AOR = 9.7; 95%CI 5.4-17.7). (See Table 5).

## Discussion

In this study the proportion of school youth engaged in oral and anal sex is considerable about 1 in 20 youth were involved in oral and anal sex practices. Moreover, a large proportion of youths involved in oral and anal sex were not taking appropriate protection measures such as consistent condom use. Reasons mentioned for having oral and anal sex included preventing pregnancy, preserving virginity, and reducing HIV and STIs transmission risks. All individual, parental, and peer level factors were associated with involvement in oral and anal sex.

Previous studies reported a wide ranging oral sex (19.6%-78%) and anal sex (5%-54%) practices [12,16,17,19]. Although the proportion of oral and anal sex in this study appears to be low, the proportion of youth engaged in multiple sexual partnerships, and the extremely low and inconsistent use of condom during such sexual encounters is a major concern. In addition, approximately 3 quarters of sexually active students in this study intend to continue having oral and anal sex in the next 6 months. This is higher than the reported 31.5% oral sex intention from America [15]. This speaks that oral and anal sexual activity among some young people is a planned experience. However, the motives behind this intention need further scrutiny. Nearly half of the currently sexually active students received gift at the exchange of oral and anal sex. Young people engaged in transactional oral and anal sex are at high risk for STIs including HIV, because they may be less able to negotiate and make decision about the timing and conditions of sex with their partners [36]. Therefore, sexual health educations need to be given about the dangers of oral and anal sex and the ways on how to protect themselves from STIs including HIV.

The results of this study highlight several key issues that merit further consideration by practitioners, teachers, parents, the community and peer educators. Since youth sexual behavior is interrelated, intertwined and influenced by a multitude of factors, intervention should target the individual, family and peer determinants rather than focusing on isolated individual behaviors.

Studies on individual level predictors of oral and anal sex are scarce. However, extant literatures on vaginal sex reported association of individual level variables such as self-esteem, college aspiration and attitude towards sex with engagement in vaginal intercourse [37-40]. Similar findings were found in this study. Low self esteem, favorable attitude towards oral and anal sex and low college aspiration were associated with involvement in oral and anal sex. This finding underscores that parents and schools should inculcate the value of education in children starting from childhood. Interventions to garner and raise the self esteem of young people as well as changing attitudes towards safe sex should be in place.

Living with both parents was protective from oral and anal sex. This concurs with the results of previous findings [19]. The possible explanation for this is families headed by two parents may have more time to supervise their children and might be physically and emotionally available to communicate about sexuality to their children than other family constellations. Therefore, marriage counseling and interventions targeting family life should be given consideration. Furthermore, maternal education was a strong predictor of oral and anal sexual intercourse. As a result, female education should be given sufficient consideration.

Consistent with other studies, best friend's sexual activity was a strong predictor for engagement in oral and anal sexual activity in this study [27,41]. Peers are main sources of information and influence related to reproductive and sexuality issues to young people [41]. As a result, correct, incorrect, safe, or risky information can be introduced, circulated and diffused among members of this social system. Thus, strengthening school peer education programme is a worthy investment to educate students about the risks associated with oral and anal sex and available protective measures.

Corroborating with previous research findings, the majority of the youth engaged in oral and anal sex wrongly perceived that these sexual acts provide protection from STIs including HIV [13,15]. This is alarming and has serious programmatic and policy implications. Unless measures are taken to change this misperception, oral and anal sex could become the source for the next wave of HIV and STIs epidemic. Therefore, the inclusion of relevant information on sexual matters and prevention of STIs in the school curriculum is essential. The majority of the youth practicing anal and oral sex also consider these modes as means of preventing pregnancy. Although that might be true, these acts do not protect against the risk of contracting STIs including HIV. Therefore, students need to be advised on safer sexual practices. Furthermore, it is vital that schools sexual health education be comprehensive enough to cover the wider sexual experiences and educate students about the risks associated with oral and anal sex.

Approximately one in seven of the sexually active student's oral sexual debut and one in five respondent's first anal intercourse in this sample occurred before the age of 10. In addition, nearly half of oral and anal sex debut of students happened without their consent. This is higher than the results of studies in different parts of Africa [42-45]. Although coerced sex may occur at any age, engagement in forced oral and anal sex at an early age, where these children are not capable to defend and protect themselves is catastrophic. Child sexual abuse is against human rights and has physical, psychological, and social consequences as well as negative impact on the education and future survival and hope of children [46]. Thus, the prevention of child sexual abuse needs investment from government, health sector, legal, education, police, the community, and the family. Prevention through public education and school health education; early detection and treatment of victims should be in place.

The major limitation of this study is the accuracy of self reported oral and anal sexual practices of respondents. As these sexual practices are considered taboo in Ethiopia there may be social desirability bias leading to under reporting. However, attempts were made to minimize this bias by using self-administered anonymous questionnaire and ensuring privacy during data collection. Despite this limitation, the large sample size and the representativeness of the sample make the findings of this study generalizable to similar population in large urban cities in Ethiopia.

## Conclusion

The findings of this study indicated that a negligible number of students initiated oral and anal sex without their consent before their teen years. Students engaged in multiple partners' oral and anal sexual relationships without proper protection, and received gift for the exchange of oral and anal sex. The majority of whom who had oral and anal sex had future intention to have oral and anal sex in the next 6 months. The main reasons for involvement in oral and anal sex were prevention of pregnancy, minimize the risk of HIV, preserving virginity and reduction of STIs transmission. Favorable attitude about oral and anal sex, low college aspirations and low self esteem were individual level predictors of oral and anal sex. Living with both parents and maternal literacy were protective from oral and anal sex. Students who perceive their best friends engage in oral and anal sex were highly likely to involve in oral and anal sexual activity.

Therefore, future interventions should target the multilevel adolescent's sexual behavior and influences at individual, parental and peer levels. Interventions that aim to illuminate educational aspirations raise the self esteem of young people, and change attitudes towards safe sex practices should be in place. School sexual health education should cover the wider sexual experience and educate students about the risks associated with oral and anal sex and its prevention mechanisms. Further in-depth exploration is needed to explain the motivation behind oral and anal sex.

## Competing interests

The authors declare that they have no competing interests.

## Authors' contributions

Both authors participated from the inception of the research idea to proposal development, data collection, analysis and preparation of the manuscript. All authors have read and approved the final manuscript.

## Pre-publication history

The pre-publication history for this paper can be accessed here:

http://www.biomedcentral.com/1471-2458/12/5/prepub
